# Modeling COVID-19 with Human Pluripotent Stem Cell-Derived Cells Reveals Synergistic Effects of Anti-inflammatory Macrophages with ACE2 Inhibition Against SARS-CoV-2

**DOI:** 10.21203/rs.3.rs-62758/v1

**Published:** 2020-08-20

**Authors:** Fuyu Duan, Liyan Guo, Liuliu Yang, Yuling Han, Abhimanyu Thakur, Benjamin E. Nilsson-Payant, Pengfei Wang, Zhao Zhang, Chui Yan Ma, Xiaoya Zhou, Teng Han, Tuo Zhang, Xing Wang, Dong Xu, Xiaohua Duan, Jenny Xiang, Hung-fat Tse, Can Liao, Weiren Luo, Fang-Ping Huang, Ya-Wen Chen, Todd Evans, Robert E. Schwartz, Benjamin tenOever, David D. Ho, Shuibing Chen, Qizhou Lian, Huanhuan Joyce Chen

**Affiliations:** The Pritzker School of Molecular Engineering, the University of Chicago; Prenatal Diagnostic Centre and Cord Blood Bank, Guangzhou Women and Children’s Medical Center, Guangzhou Medical University; Department of Surgery, Weill Cornell Medicine; Department of Surgery, Weill Cornell Medicine; The Pritzker School of Molecular Engineering, the University of Chicago; Department of Microbiology, Icahn School of Medicine at Mount Sinai; Aaron Diamond AIDS Research Center, Columbia University Irving Medical Center; Department of Medicine, Li Ka Shing Faculty of Medicine; the University of Hong Kong; Department of Medicine, Li Ka Shing Faculty of Medicine; the University of Hong Kong; Prenatal Diagnostic Centre and Cord Blood Bank, Guangzhou Women and Children’s Medical Cent; Sandra and Edward Meyer Cancer Center, Department of Medicine, Weill Cornell Medicine; Genomic Resource Core Facility, Weill Cornell Medicine; Genomic Resource Core Facility, Weill Cornell Medicine; Genomic Resource Core Facility, Weill Cornell Medicine; Department of Surgery, Weill Cornell Medicine; Genomic Resource Core Facility, Weill Cornell Medicine; Department of Medicine, Li Ka Shing Faculty of Medicine; the University of Hong Kong; Prenatal Diagnostic Centre and Cord Blood Bank, Guangzhou Women and Children’s Medical Center, Guangzhou Medical University; Department of Pathology, The Second Affiliated Hospital of Southern University of Science and Technology; Institute for Advanced Study (IAS), Shenzhen University; Department of Medicine, Hastings Center for Pulmonary Research, Division of Pulmonary, Critical Care and Sleep Medicine; Department of Surgery, Weill Cornell Medicine; Division of Gastroenterology and Hepatology, Department of Medicine, Weill Cornell Medicine; Department of Microbiology, Icahn School of Medicine at Mount Sinai; Department of Microbiology, Icahn School of Medicine at Mount Sinai; Department of Surgery, Weill Cornell Medicine; Department of Medicine, Li Ka Shing Faculty of Medicine; the University of Hong Kong; The University of Chicago

**Keywords:** COVID-19, SARS-CoV-2, Human pluripotent stem cell, Macrophages, disease modeling

## Abstract

Dysfunctional immune responses contribute critically to the progression of Coronavirus Disease-2019 (COVID-19) from mild to severe stages including fatality, with pro-inflammatory macrophages as one of the main mediators of lung hyper-inflammation. Therefore, there is an urgent need to better understand the interactions among SARS-CoV-2 permissive cells, macrophage, and the SARS-CoV-2 virus, thereby offering important insights into new therapeutic strategies. Here, we used directed differentiation of human pluripotent stem cells (hPSCs) to establish a lung and macrophage co-culture system and model the host-pathogen interaction and immune response caused by SARS-CoV-2 infection. Among the hPSC-derived lung cells, alveolar type II and ciliated cells are the major cell populations expressing the viral receptor ACE2 and co-effector TMPRSS2, and both were highly permissive to viral infection. We found that alternatively polarized macrophages (M2) and classically polarized macrophages (M1) had similar inhibitory effects on SARS-CoV-2 infection. However, only M1 macrophages significantly up-regulated inflammatory factors including IL-6 and IL-18, inhibiting growth and enhancing apoptosis of lung cells. Inhibiting viral entry into target cells using an ACE2 blocking antibody enhanced the activity of M2 macrophages, resulting in nearly complete clearance of virus and protection of lung cells. These results suggest a potential therapeutic strategy, in that by blocking viral entrance to target cells while boosting anti-inflammatory action of macrophages at an early stage of infection, M2 macrophages can eliminate SARS-CoV-2, while sparing lung cells and suppressing the dysfunctional hyper-inflammatory response mediated by M1 macrophages.

## Introduction

The infection of severe acute respiratory syndrome coronavirus 2 (SARS-CoV-2) has already caused more than 22.4 million Coronavirus Disease-2019 (COVID-19) cases internationally (https://coronavirus.jhu.edu/map.html). Most COVID-19 patients show mild to moderate symptoms of fever, dry cough, fatigue and diarrhea, however, approximately 15% of confirmed cases progress to severe pneumonia, acute respiratory distress syndrome (ARDS) or multi-organ failure (Guan et al., 2020). The progression from mild to severe disease or death is principally attributed to dysfunctional immune responses (Mehta et al., 2020; Wang et al., 2020) together with viral damage of target cells. Given the lack of an effective vaccine or medication, a thorough understanding of immunological features caused by SARS-CoV-2 is critically important for studying viral pathobiology and therapeutic development.

Alveolar macrophages (AMs) are key sentinel cells for host defense in the respiratory system, producing cytokines and chemokines that are crucial components of innate immunity and mediators of immunopathology (Allard et al., 2018). The polarization of macrophages confers a heterogeneous function and plasticity depending on the duration of stimulation and microenvironment, which are discrete phenotypes associated with different inflammatory responses, typically termed the M1φ /pro-inflammatory and M2φ /anti-inflammatory macrophages (Gomez Perdiguero et al., 2015; Wynn et al., 2013). The distinction is known to be over-simplified, with macrophage dynamic activities spread along the M1–M2 phenotypic spectrum(Bian, 2020; Shapouri-Moghaddam et al., 2018). However, in general, M1φ destroys pathogens by producing a large number of pro-inflammatory cytokines such as IL-1β, TNFα, IL-6 and IL18. In contrast, M2φ exhibits higher activity in phagocytosis against pathogens and for anti-inflammation (Mills, 2015; Murray, 2017). Recent studies (Liao et al., 2020; Xu et al., 2020) on immunity of COVID-19 patients indicate that the cells damaged by SARS-CoV-2 infection induced innate inflammation in the lungs that is largely mediated by pro-inflammatory macrophages and granulocytes. In addition to local damage, the pro-inflammatory macrophages release cytokines/chemoattractants and prime adaptive immune cell responses, which in some cases lead to dysfunctional immune responses and cytokine storm, followed by respiratory and even multi-organ failure (Xu et al., 2020). These studies imply a crucial role for macrophages in the progress of SARS-CoV-2 infection; a deeper understanding of the interactions among targeted cells, macrophages and SARS-CoV-2 could offer new ideas to help combat this deadly contagious disease.

The current most widely used model for SARS-CoV-2 research is the African green monkey derived Vero cells, which are very limited for modeling human disease. Although primary macrophages are more functionally or phenotypically representative of native macrophages in the tissue from which they are derived, they are difficult to obtain, proliferate slowly, and are often poorly characterized (Jobe et al., 2017). In this study, we generated lung cells and macrophages paired from the same cell origin, human pluripotent stem cell (hPSC) lines. This strategy overcomes a common concern about histocompatibility when studying human immune cells with other cell types, and provides theoretically unlimited cell resources for reliably modeling and studying immunology of macrophages and human lungs during SARS-CoV-2 infection. Our results using this platform demonstrate a potential therapeutic strategy through a combination of boosting anti-inflammatory macrophages and intervention of viral entry, to control SARS-CoV-2 infection at the immune defense-based protective phase while circumventing the inflammation-driven damaging phase.

## Results

### Macrophage involved at the severe stage of COVID-19

**Figure 1:**
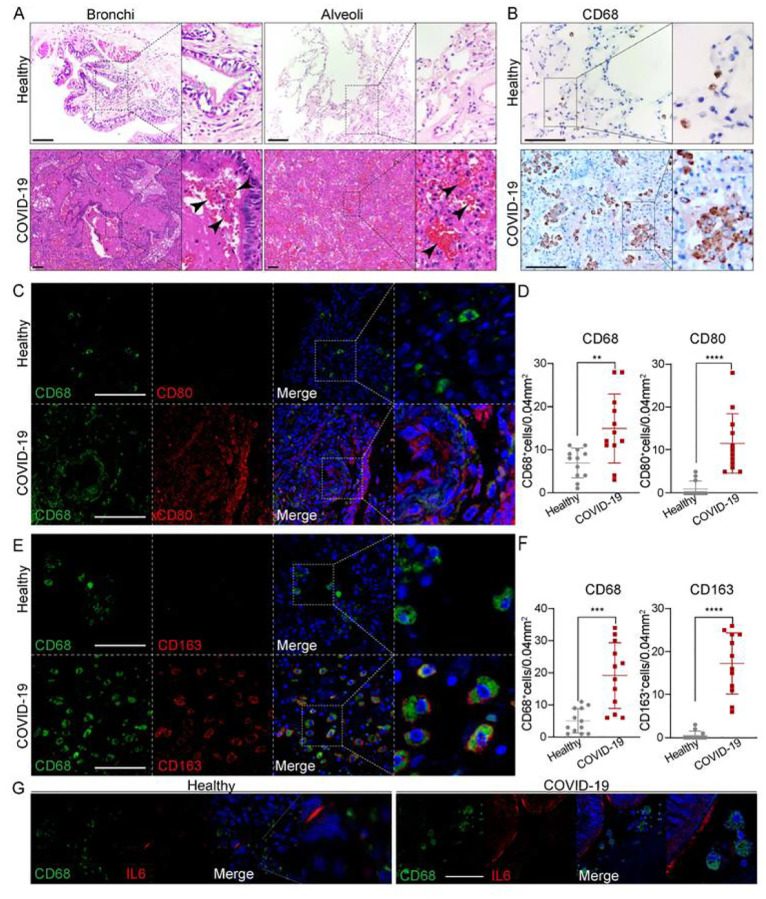
Macrophages were highly involved at the severe stage of COVID-19(A) H+E (Hematoxylin and Eosin) staining on the bronchial or alveolar region in healthy or severe COVID-19 case. Pulmonary hemorrhagic infarct (denoted by arrowheads) (B) Immunohistochemistry (IHC) using antibody against CD68 revealed macrophage with aggregated phenotype and enlarged nuclei in COVID-19 lung, compared to the ones in the healthy lung. (C) Immunofluorescence (IF) staining on healthy or COVID-19 distal lung tissues using antibodies against CD68 (pan-macrophage marker), and CD80 (M1 macrophage marker) (D) Quantification on CD68+ or CD80+ macrophages in healthy or COVID-19 distal lung tissues. (E) IF staining on healthy or COVID distal lung tissues using antibodies against CD68 and CD163 (M2 macrophage marker) (F) Quantification on CD68+ or CD163+ macrophages in healthy or COVID-19 distal lung tissues. (G) IF staining on healthy or COVID-19 distal lung tissues using antibodies against CD68 and IL-6. Scale bar = 100 μm in all images in Figure 1. Data were presented as mean ± STDEV. P values were calculated by unpaired two-tailed Student’s t-test. **P < 0.01, ***P < 0.001, and ****P < 0.0001.

To better understand how macrophages impact COVID-19 progression, we compared immune cells and inflammatory factors in lung tissues obtained from autopsies of COVID-19 patients or healthy donors. First, histological changes in lung tissues from COVID-19 patients were examined. Compared to healthy lung tissues, this revealed extensive necrotizing bronchiolitis with necrotic bronchial epithelial cells and severe alveolitis with atrophy and desquamation, displayed in the lumen of the patient’s lung (Figure 1A). Of note, pulmonary hemorrhagic infarct with abundant inflammatory infiltration (arrow heads) were extensively present through the whole alveoli and bronchial regions (Figure 1A). Recently, it was reported that proinflammatory FCN^+^ monocyte-derived macrophages were mainly present and FABP4^+^ alveolar macrophages were greatly reduced in the bronchoalveolar lavage fluid from patients with severe COVID-19, whereas mild and moderate cases were characterized by the presence of highly clonally expanded CD8^+^ T cells(Liao et al., 2020). Therefore, we examined if macrophages were dominantly present in the diseased patient’s lung. Immunostaining against pan macrophage marker CD68 showed abundant macrophages were extensively distributed through the whole lung tissue with aggregated phenotypes (Figure 1B), in agreement with the above-cited report. However, macrophages are multifaceted and distinct functions of macrophages highly depends on polarization, characterized generally as M1/pro-inflammatory or M2/anti-inflammatory macrophages. We thus further examined M1 macrophage marker CD80, and M2 macrophage marker CD163 (Figures 1C–F). The results revealed that cells positive for either CD80 or CD163 were both aberrantly represented in the patient’s lung tissue (Figures 1 C–F). Indeed, CD68^+^, CD80^+^ and CD163^+^ macrophage populations were significantly expanded in the patient’s lung tissue, suggesting expansion of both M1 and M2 macrophage populations in severe disease. We also examined several cytokines that are mainly produced by macrophages and found key pro-inflammatory cytokine IL-6 was intensively expressed in the lumen of the patient’s lung tissue (Figure 1G). Taken together, the data supports a need to further examine the roles of M1 and M2 macrophages in COVID-19 progression.

### Co-culture of lung cells and macrophages derived from hPSCs

To further investigate the interaction among macrophages, lung cells and SARS-CoV-2, we established a co-culture model using cells derived from the same hPSC line (RUES2 or H1), which provide a genetically defined background for immune study. Several effective methods have recently been described (Dye et al., 2015; Hurley et al., 2020; Mou et al., 2012) for generating major cell types found in human lung tissues by directed differentiation of hPSCs using growth factors and chemicals to alter cell fate determining signaling pathways. Based on a protocol modified from ones previously developed by our lab and others (Chen et al., 2019; Huang et al., 2015; Huang et al., 2014), hPSCs were differentiated in a stepwise approach into definitive endoderm (DE), followed by anterior foregut endoderm (AFE), lung progenitor cells (LPs), and finally by specification to bronchial and alveolar lineage cells (Figures S1A–D, S2A–B). Single cell transcriptomic profiling identified in the cultures many expected populations that comprised 6 main clusters, including alveolar type II (AT2) cells (SP-B^+^, SP-C^+^, SP-D^+^, ABCA3^+^), alveolar type I (AT1) cells (AGER^+^AQP5^+^), ciliated cells (FOXJ1^+^CAPS^+^), stromal cells (DCN^+^), club cells (SCGB3A1^+^SCGB3A2^+^), as well as low numbers of goblet cells (MUC5AC^+^MUC5B^+^), basal progenitor cells (P63^+^), and neuroendocrine cells (ASCL1^+^CALCA^+^) (Figures S3A–D). ACE2, the putative SARS-CoV-2 receptor, and TMPRSS2, the co-effector for viral entry (Hoffmann, 2020), were detected in AT2, AT1 and ciliated cells, in clusters 0, 2, 3 (Figures S3E–F). The immunostaining results further validated that ACE2 is mainly co-expressed with SP-B or pro-SP-C in AT2 cells, and FOXJ1 in ciliated cells (Figure S2B), consistent with results previously reported in primary human lung tissues(Ziegler, 2020).

**Figure 2:**
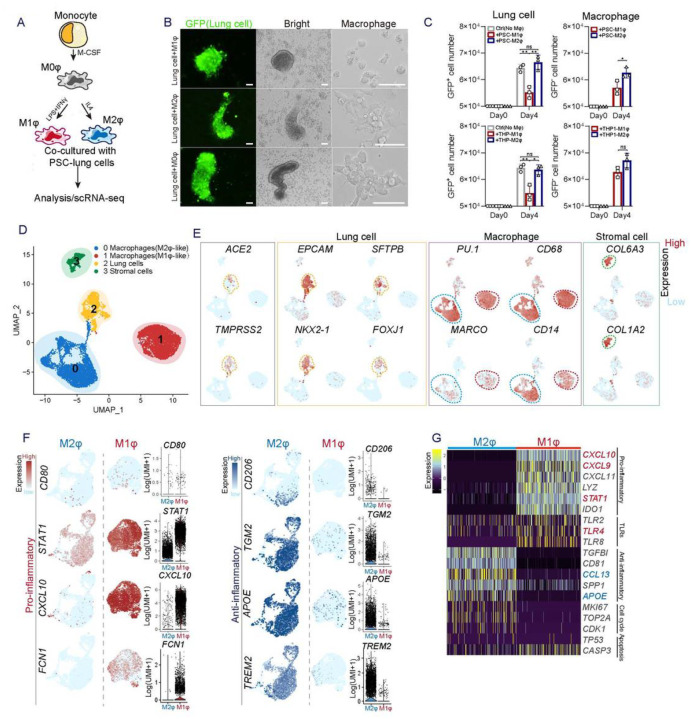
Characterization of the co-culture of lung cells and macrophages derived from hPSCs (A) Schematic of the experimental flowchart on the co-cultures. (B) Representative bright or fluorescence images of the co-culture of lung cells and macrophages derived from hPSC line RUES2. Lung cells are GFP positive. Scale bar = 50 μm (C) Quantification of lung cells, and macrophages in the co-cultures of lung cells and iMφ, iM2φ or 293T cells. P values were calculated by unpaired two-tailed Student’s t-test. *P < 0.05, **P < 0.01. (D) UMAP of scRNA seq on the two-co-cultures (lung and iM1φ co-culture; lung and iM2φ co-culture). Colored and annotated with cluster 0–3 representing iM2φ, iM1φ, lung epithelial cells and stromal cells. (E) ACE2, TMPRSS2, as well as putative cell-fate related markers differentially expressed in each cluster in UMAPs. Relative expression of each marker gene range from low (light blue) to high (pink) as indicated. Individual cell positive for each marker are donated by red dots. The main population of ACE2 or TMPRSS2 positive populations are circled in the dotted line. (F) A set of pro-or anti-inflammatory factors or cell-fate related markers differentially expressed in the cluster of iM1φ or iM2φ in UMAPs. Relative expression of each marker gene range from low (light blue) to high (pink) as indicated. The violin plot shows the expression level (log2(UMI+1)) of the indicated gene in each cluster. (G) Heatmap presenting top differential expression genes related to pro- or anti-inflammatory factors, Toll-like receptors (TLRs), cell cycle regulation or apoptosis, in iM2φ or iM1φ.

To generate macrophages and monocytes from hPSCs we used protocols published by us and others (Buchrieser et al., 2017; Cao et al., 2019; Duan et al., 2018; Lachmann et al., 2015; Lang et al., 2018). In brief, the hPSCs were first induced to mesoderm and then to vascular mesoderm cells, which were further differentiated to hematopoietic stem and progenitor cells (HSPCs). The HSPCs were induced to differentiate into functional macrophages by treatment with monocyte cytokines IL3 and Macrophage-colony stimulating factor (M-CSF) (Figures S4A–C). The hPSC-derived macrophages expressed major macrophage/monocyte markers such as CD14, CD11b and CD68 (Figure S4D) and were readily polarized to CD68^+^CD206^+^ macrophages, or CD68^+^FCN1^+^STAT1^hi^ macrophages (Figures 2A, D–H, Figure S5B) upon stimulation by IL-4 or IFNγ and lipopolysaccharide (LPS) respectively (Figure S5A). Very few iMφs expressed ACE2 and TMPRSS2 based on single cell RNA (scRNA) profiling (Figure S5B).

Next, the hPSC-induced lung cells (iLung) and macrophages (iMφ) were plated and cultured together in a 1:1 ratio (Figure 2A), similar to the ratio of lung cells and macrophages in distal bronchial or alveolar regions in human lung (Kyle J. Travaglini, 2020). The iLung was derived from the hPSC lines carrying a Doxycycline-inducible GFP reporter gene, which allowed the distinction of iLung and iMφ in live cultures (Figure 2B). A significantly lower number of GFP^+^ iLung were observed after four-day co-culture with iMφ of M1 phenotype (iM1φ), than seen in the co-culture with iMφ of M2 phenotype (iM2φ) or control 293T cells (Figure 2C). The scRNA profiles further revealed decreased expression of proliferation-associated genes *MKI67and TOP2A* and increased expression of apoptosis-related genes *TP53, CASP3, BAX, MCL1*, in the iLung co-cultured with iM1φ, but not in co-cultures with iM2φ (Figure S6D). These results were in alignment with the phenotype of pro-inflammatory activities of iM1φ, as scRNA-seq data detected a set of pro-inflammatory factors, *IL1B, IL18, STAT1, FONT, CXCL9, CXCL10, CXCL11, CXCL16, CCL2* highly expressed in iM1φ (Figure 2F–G, S5B–C). In contrast, iM2φ mainly expressed anti-inflammatory factors or immunoregulatory genes such as *TGM2, APOE, A2M, CCL13, CCL26* and *TREM2* (Figure 2F–G, S5B–C). Gene Ontology (GO) enrichment analysis comparing iM1φ and iM2φ revealed over-activation of differential signaling pathways such as pro-inflammatory IFNγ, type I IFN, and neutrophil activation in iM1φ; anti-inflammatory and tissue damage-repair process of RNA catabolic process, protein co-localization to endoplasmic reticulum in iM2φ (Figures S6B, C). Similar phenotypes were observed in the iLung co-cultured with THP-1, an established monocyte line, upon activation of M1 or M2 phenotype (Figure 2C). The results indicate that activation of M1-macrophage was sufficient to create a toxic environment for the iLung even in the absence of viral infection.

### Immune response of macrophages following SARSCoV-2 infection

**Figure 3:**
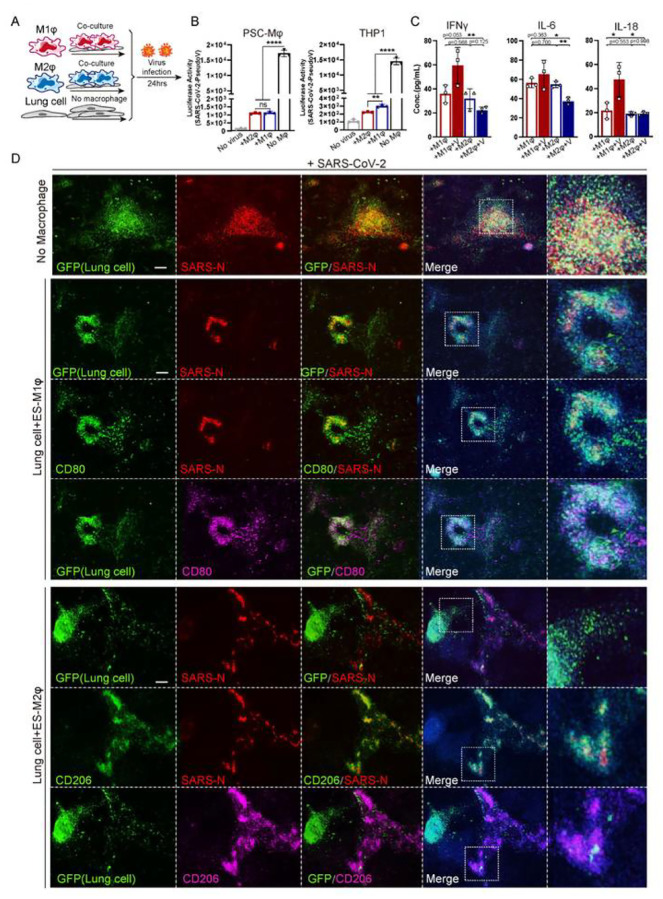
The effects of M1 or M2 macrophages on SARS-CoV-2 infection (A) Schematic of the experimental flowchart on the co-cultures. (B) Luciferase activity of the co-cultures of lung cells and M1, M2 macrophages (iMφ or THP-1) or 293T cells (control) at Mock or infected with SARS-CoV-2 pseudo-entry virus at 24 hpi (MOI=0.01). P values were calculated by unpaired two-tailed Student’s t test. **P < 0.01, ****P < 0.0001. (C) Quantification of inflammatory factors in the co-culture medium at Mock or infected with SARS-CoV-2 pseudo-entry virus at 24 hpi (MOI=0.01). P values were calculated by Chi-Square Test. *P < 0.5, **P < 0.01. (D) IF staining on the co-cultures of iLung cells and iM1φ, iM2φ, or 293T, at Mock or infected with SARS-CoV-2 virus at 24 hpi (MOI=0.01), using antibodies detecting SARS-N protein, CD80 or CD206. ILung cells expressed GFP. Scale bar = 100 μm

To model the immune response of macrophages to SARS-CoV-2 infection on lung cells, virus was introduced to the co-culture system (Figure 3A). As a first step to measure effects of macrophages on viral entry into lung cells, we used a SARS-CoV-2 pseudo-entry virus, in which the backbone of a VSV-G pseudo-typed ΔG-luciferase virus carries the SARS-CoV-2 spike protein incorporated in the surface of the viral particle (Nie et al., 2020; Whitt, 2010). High luciferase activity was readily detected in iLung 24 hours after viral infection at MOI=0.01, but not in iMφ or 293T in the co-culture (293T cells were used as a co-culture control, based on our preliminary data and previous report that the permissiveness of 293T to SARS virus is low (Wenhui Li, 2003)) (Figure 3B), and immunostaining confirmed that the viral luciferase protein was co-localized with ACE2^+^ cells in the iLung cultures (Figure S7B). Since the luciferase gene was expressed after the virus entered host cells, the luciferase activity correlated to the amount of viral entry host cells. Luciferase activity was markedly decreased in the co-cultures of iLung with all three lines of macrophages, iMφ, THP-1 and U937; no significant difference was found between hPSC-derived iMφ or iM2φ, indicating they have the similar inhibitory effects on viral infection (Figure 3B, Figure S7A). The results were further validated by immunostaining study that substantial decrease of luciferase protein was detected in iLung cells co-cultured with iMφ, compared to those co-cultured with 293T (Figure S7A). The potential of iMφ to inhibit viral replication and spreading was next studied by infection with a patient-derived SARS-CoV-2 virus in the co-cultures. After 24 hours incubation with the SARS-CoV-2 virus (USA-WA1/2020, MOI=0.01), a significant decrease of viral protein was observed in the co-culture of iLung and iMφ, compared to the co-culture of iLung and 293T. Strikingly, most SARS-CoV-2 virus SARS-N protein was detected in the M2-iMφ when co-cultured with iLung, while in contrast, substantial levels of SARS-N protein was detected in iLung cells in the co-cultures using M1-iMφ or 293T (Figure 3D). The findings suggest that phagocytosis activity of M2-iMφ functioned as protection for iLung from viral infection.

**Figure 4:**
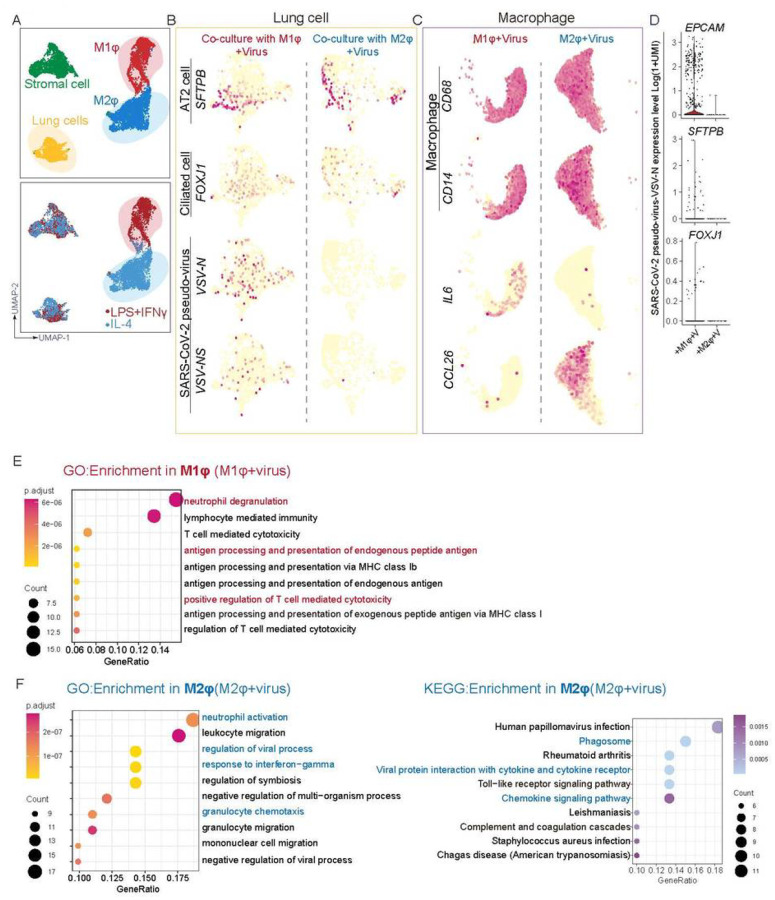
scRNA analysis of iM1φ, iM2φ or iLung upon viral infection (A) UMAP of scRNA seq on the SARS-CoV-2 pseudo-virus infected co-cultures (lung and iM1φ co-culture; lung and iM2φ co-culture). Colored and annotated with 4 clusters representing iM2φ, iM1φ, lung cells and stromal cells. (B) SARS-CoV-2 pseudo-virus genes, and putative cell-fate related markers differentially expressed in iLung cells co-cultured with iM1φ or iM2φ in UMAPs. Relative expression of each marker gene range from low (light yellow) to high (red) as indicated. Individual cells positive for each marker are denoted by red dots. (C) Inflammatory factors (IL6 and CCL26), and putative cell-fate related markers(CD14 and CD68) differentially expressed in iM1φ or iM2φ co-cultured with ilung in UMAPs. Relative expression of each marker gene range from low (light yellow) to high (red) as indicated. Individual cells positive for each marker are denoted by red dots. (D) The violin plot shows the expression level (log2(UMI+1)) of SARS-CoV-2 pseudo-virus specific genes in each cluster. (E) GO enrichment analysis in iM1φ. Important pathways related to immune response or viral reaction is highlighted in red (iM1φ) (F) GO and KEGG enrichment analysis in iM2φ. Important pathways related to immune response or viral reaction is highlighted in blue (iM2φ)

Several approaches were taken to thoroughly examine the immune response following iMφ on SARS-CoV-2 infection. First, a cohort of cytokines and inflammatory factors that are known to be important for innate or adaptive immune responses were profiled, in the culture medium 24 hours after infection with the SARS-CoV-2 pseudo virus. Increased levels of IFNγ, IL-6, and IL-18 were found in the co-cultures of iLung with M1-iMφ, while these were decreased in the co-cultures of iLung with M2-iMφ (Figure 3C). To further characterize at the transcriptomic level the response of iLung and iMφ following viral infection, scRNA-seq was performed on the co-cultures with SARS-CoV-2 pseudo virus infection and the analysis revealed that a set of anti-inflammatory factors and anti-viral activity related genes, such as *CCL26, CCL13, ISG15, IFITM2 and IFITM3,* were clearly upregulated when cultures contained M2-iMφ (Figure 4A and C, Figures S8A). In contrast, pro-inflammatory factors, such as *IL-6, S100A8/A9, LYZ* and *TLR4* were highly expressed when the cultures contained M1-iMφ. (Figure 4A, C, Figures S8A). Gene enrichment analysis comparing iM1φ and iM2φ revealed over-activation of differential signaling pathways such as neutrophil degranulation and antigen processing and presentation, regulation of T cell mediated cytotoxicity in iM1φ; granulocyte chemotaxis, response to interferon-gamma as well as phagocytosis in iM2φ (Figures 4 E and F). Moreover, IL10 signaling related genes such as *IL10RA, IL10RB, STAT3, SOCS3, TIMP1 and IRS2* were enriched in iM2φ, suggestive of anti-inflammatory macrophages (Figure S8D). The above results demonstrate a differential immune response of iM2φ versus iM1 φ upon viral entry into host cells, as iM2φ increased phagocytosis activity and released anti-inflammatory factors, while iM1φ increased antigen-presenting activity and released pro-inflammatory factors.

Correlating with the above phenotypes, up-regulation of cell growth arrest or death-related genes, such as *GAS6, BTG2, PDCD6, CCAR1, TP53I11, TP53INP1,* and activation of programmed death signaling pathways as well as higher mitochondrial genes, *MT-CYB, MT-CO1, MT-CO2, MT-ND1* (Figure S8B and C), were detected in the co-cultures with iLung with iM1φ , but not with iM2φ (Figure S8B). Previous studies by us (Yuling Han, 2020) and others (Conti et al., 2020) suggested that lung cells display self-immune defense after SARS-CoV-2 infection, releasing proinflammatory factors, such as *CXCL2, CCL2, CXCL3* and *IL1A,* as well as *BCRC3, AADAC,* and *ATPB4.* The GO and KEGG analysis in our current co-culture based data suggest that upregulation in pathway networks including leukocyte chemotaxis NF-κB signaling, IL-17 signaling, viral protein interaction with cytokine-cytokine receptor, and response to type I interferon, combined with the pro-inflammatory reaction of M1 macrophages, could lead to further pulmonary inflammation and damage (Figure S8E). Moreover, the scRNA-seq profiling data further validated the immunostaining results showing that few if any iLung cells in the co-culture with M2-iMφ displayed detectable viral gene expression, in contrast to a significantly higher number of iLung cells in the co-culture with M1-iMφ (Figure 4B). Most infected AT2 cells and ciliated cells were also found in the co-culture with M1-iMφ, indicating a stronger protective effect on iLung cells by M2-iMφ (Figure 4D). Altogether, these findings suggest that activation of pro-inflammatory macrophages can aggravate lung cell damage, beyond the destruction by viral infection; in contrast, activation of anti-inflammatory macrophages provides a protective effect for lung cells from viral infection.

### Blockage of ACE2 enhances elimination of SARS-CoV-2 by macrophages

**Figure 5:**
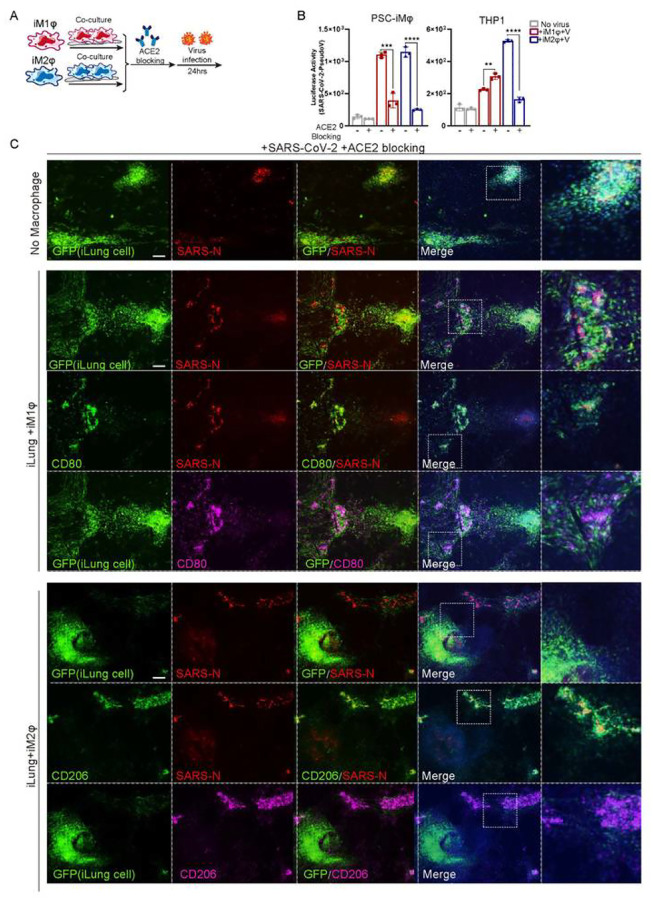
The effects of macrophages in combination with ACE2 blockage on SARS-CoV-2 infection (A) Schematic of the experimental flowchart on the co-cultures. (B) The ACE2 blockage antibody was applied two hours prior to the virus presence, and the luciferase activity of the co-cultures of lung cells and M1, M2 macrophages (iMφ or THP-1) or 293T cells (control) was measured at Mock or infected with SARS-CoV-2 pseudo-entry virus at 24 hpi (MOI=0.01). P values were calculated by unpaired two-tailed Student’s t-test. ***P < 0.001, ****P < 0.0001. (C) The ACE2 blockage antibody was applied two hours prior to the virus presence, IF staining was performed on the co-cultures of iLung cells and iM1φ, iM2φ, or 293T, at Mock or infected with SARS-CoV-2 virus at 24 hpi (MOI=0.01), using antibodies detecting SARS-CoV-2 NSP14 protein, CD80 or CD206. ILung cells expressed GFP Scale bar = 100 μm

Several studies (Tay et al., 2020) on mild or recovered COVID-19 cases indicated that in a healthy immune response, neutralizing antibodies produced in these individuals can block viral infection, followed by alveolar macrophages recognizing the neutralized viruses and clearing them by phagocytosis. We sought to model this process using an ACE2 blocking antibody to inhibit virus entry to target cells, thus decreasing the viral loads (Figure 5A), to test if this enhances phagocytosis activity of macrophages. As expected, incubation with ACE2 blocking antibody two hours prior to infection of SARS-CoV-2 pseudo virus, reduced markedly the luciferase activities in co-cultures of iLung with either M1 or M2-iMφ, although the decrease of luciferase signal was most pronounced in the co-cultures with M2-iMφ (Figure 5B, Figure S9A). Immunostaining results validated that luciferase protein dramatically decreased in the iLung cells co-cultured with iMφ, compared to those co-cultured with 293T (Figure S9A).

Similarly, immunostaining results from the experiments performed using SARS-CoV-2 virus further revealed that that most SARS-CoV-2-N protein was found in the M2-iMφ, but not in the iLung cells, while the N protein was clearly found in iLung cells in the co-culture with M1-iMφ or 293T (Figure 5C). These results demonstrated that an early intervention of viral infection by blocking ACE2 in target cells can increase the clearance of virus by macrophages, especially synergizing with the phagocytosis activity of M2-macophages to further provide protection for target cells and reduce the damage by inflammatory factors produced by M1-macrophages.

## Discussion

The study of human host-immune systems with pathogens has depended historically on the use of animal models, largely due to limited cell resources derived from human tissues. Immune research on COVID-19 is limited by the types of models available for study. Recently, a transgenic mouse strain(McCray et al., 2007) has been made with human ACE2 expression regulated by human cytokeratin-18 promoter, but the ACE2 expression in human is more complex than that in the mice. Another model is ferret(Kim et al., 2020), which can be infected with SARS-CoV-2, but does not develop hyper-inflammation in the lung. Recent advances in stem cell biology, especially the technology to differentiate human pluripotent stem cells (hPSCs) into functional immune cell types, provide a rigorous human system for studying immunology and therapeutics. In this report, we describe a new cell co-culture system in which the immune cells, specifically monocytes/macrophages, and lung lineage cells are produced by directed differentiation of hPSCs. Several key features make the human cell model an ideal system for studying immunology of SARS-CoV-2. The model contains the host cells and immune cells from the same hPSC lines, avoiding concern of histocompatibility, while it can provide abundant numbers of cells with a genetically defined background for robust mechanistic or therapeutic studies.

The innate immune response mainly mediated by macrophages or granulocytes, responding to tissue damage caused by SARS-CoV-2 infection, likely contributes to acute respiratory distress syndrome (ARDS) that is characterized by the rapid onset of widespread inflammation in lung and subsequent respiratory failure (Xu et al., 2020). Our study in COVID-19 patient samples validated a correlation of macrophages and the disease, showing a heavy infiltration of pro-inflammatory macrophages in tissue samples from distal lung regions with high levels of inflammatory cytokine IL-6 in the severe cases. The macrophage and lung cell co-culture model combined with single cell transcriptomics was then applied to interrogate the differential immune responses of pro- or anti-inflammatory macrophages following SARS-CoV-2 infection. We discovered that pro- and anti-inflammatory macrophages both have similar capacity to eliminate the virus in the context of a moderate viral load. However, the immune reaction of pro-inflammatory macrophages following SARS-CoV-2 pseudo-virus infection led to more damage on lung cells and secretion of a set of inflammatory factors including IL6, IL18 and CXCL10 that are known to be mediators in dysfunctional immune responses and cytokine release syndrome (CRS). In contrast, anti-inflammatory macrophages protected lung cells from viral infection, and diminished pulmonary inflammation by phagocytosis and production of anti-inflammatory factors related in IL10 signaling. Finally, inhibiting viral entry in target cells using an ACE2 blocking antibody, diminished viral infection and enhanced the elimination of viruses. In particular, the intervention on viral entry can synergize with the phagocytosis and antiviral activity of macrophages, resulting in a more pronounced clearance of virus and protection of target cells.

The immune responses induced by SARS-CoV-2 infection can be clinically divided into two phases, the first immune defense-based protective phase and the second inflammation-driven damaging phase (Shi et al., 2020; Tay et al., 2020). Boosting immune responses in the first phase of the viral incubation while suppressing it in the second phase, could help eliminate the virus and preclude disease progression to severe stages. Our study demonstrates the potential and significance of anti-inflammatory macrophages for inhibiting viral infection and protecting host cells. Furthermore, several ACE2 blocking antibodies, or virus neutralizing antibodies (Long Chen, 2020) have been reported as currently under development or testing in clinical trials to treat COVID-19 patients (https://clinicaltrials.gov/ct2/show/NCT04335136). On the other hand, cell-based immunotherapy using autologous or stem cell-derived macrophage has been studied or tested in clinical trials (https://doi.org/10.1186/ISRCTN10368050) to treat a variety of human diseases, especially the application of anti-inflammatory macrophages in treating chronic inflammatory diseases(Moroni et al., 2019) (Chan and Viswanathan, 2019; Rodell et al., 2019). With these resources and information on hand, the strategy we developed in this study, combining blocking ACE2 receptor while boosting anti-inflammatory macrophages, provides some new ideas that can be employed to help combat this deadly contagious disease.

## Materials And Methods

### METHOD DETAILS

#### Patient’s lung tissues

The paraffin-embedded lung tissues were acquired from the department of pathology in the 3^rd^ hospital of Shenzhen. They recently reported the pathological changes of lungs from a 66-year-old male died in critical COVID-19 infection^2^. The patient developed respiratory failure and septic shock during the treatment and was done with transplant. Informed consent was obtained from the patient and family. The diagnosis of COVID-19 pneumonia was based on the “Coronavirus Pneumonia Prevention and Control Plan” (7th edition) newly issued by the National Health Commission, China ^3^. Nasopharyngeal swabs were collected and COVID-19 was detected by real-time polymerase chain reaction. Infection was defined as at least two positive test results. Surgical informed consent was obtained and the study was approved by IRB in the third People’s hospital of Shenzhen.

#### hPSC lung differentiation

Protocols for maintenance of hPSCs and generation of lung cells were slightly modified from previous studies ^4,5^. The hESC line-RUES2 or H1 was cultured on irradiated mouse embryonic fibroblasts (Global Stem, cat. no. GSC-6001G) at a density of 20,000–25,000 cells/cm^2^ in a medium of DMEM/F12, 20% knockout serum replacement (Life Technologies), 0.1 mM β-mercaptoethanol (Sigma Aldrich) and 20 ng/ml bFGF (R&D Systems), and medium was changed daily. hESC cultures were maintained in an undifferentiated state at 37 °C in a 5% CO2/air environment until stem cells reached about 90% confluence.

hESC differentiation into endoderm was performed in serum-free differentiation (SFD) medium of IMDM/F12 (3:1) (Life Technologies) supplemented with N2 (Life Technologies), B27 (Life Technologies), 50 μg/ml ascorbic acid, 2 mM Glutamax, 0.4 μM monothioglycerol, 0.05% BSA at 37 °C in a 5% CO_2_/5% O_2_/95% N_2_ environment. hESCs were treated with Accutase and plated onto low attachment 6-well plates (Corning Incorporated, Tewksbury MA), resuspended in endoderm induction medium containing 10 μM Y-27632, 0.5 ng/ml human BMP-4, 2.5 ng/ml human bFGF, 100 ng/ml human Activin A, for 72–76 hours dependent on the formation rates of endoderm cells. On day 3, the endoderm bodies were dissociated into single cells using 0.05% Trypsin/0.02% EDTA and plated onto fibronectin-coated, 24-well tissue culture plates (~100,000–150,000 cells/well). For induction of anterior foregut endoderm (AFE), the endoderm cells were cultured in SFD medium supplemented with 1.5 μm Dorsomorphin dihydrochloride (R&D Systems) and 10 μM SB431542 (R&D Systems) for 48 h, and then switched to 24 h of 10 μM SB431542 and 1 μM IWP2 (R&D Systems) treatment. For induction of early stage lung progenitor cells (day 6–15), the resulting anterior foregut endoderm was treated with 3 μM CHIR99021, 10 ng/ml human FGF10, 10 ng/ml human FGF-7, 10 ng/ml human BMP-4 and 50–60nM all-trans retinoic acid (ATRA), in SFD medium for 8–10 d. The day 10–15 cultures were maintained in a 5% CO_2_/air environment. On days 15 and 16, the lung progenitor cells were replated after one minute trypsinization onto fibronectin-coated plates, in the presence of SFD containing 3 μM CHIR99021, 10 ng/ml human FGF10, 10 ng/ml human FGF7, in a 5% CO_2_/air environment. For differentiation of mature lung cells (day 25 to 55), cultures were re-plated after brief trypsinization onto 3.3% Matrigel-coated 24-well plates in SFD media containing maturation components containing 3 μM CHIR99021, 10 ng/ml human FGF-10; 10 ng/ml human FGF-7, and DCI (50 nM Dexamethasone, 0.1 mM 8-bromo-cAMP (Sigma Aldrich) and 0.1 mM IBMX (3,7-dihydro-1-methyl-3-(2-methylpropyl)-1H-purine-2,6-dione) (Sigma Aldrich)). The protocol details are summarized in Figure S1A.

All embryonic stem cell studies were approved by the Institutional Review Board (IRB) at the University of Chicago, or by the Tri-Institutional ESCRO committee (Weill Cornell Medicine, Memorial Sloan Kettering Cancer Center, and Rockefeller University).

#### hPSC macrophage differentiation

We derived macrophages from hESC line H1 or RUES2 and adapted based on previously reported protocols^6–8^. For macrophage differentiation, at day −2, hESCs were digested into single-cell suspension by 1 mg/ml Accutase (Stemcell Technologies) and plated onto Matrigel-coated culture dishes at a density of 2× 10^4^ cells/cm^2^ in mTeSR1 medium with 5uM Y27632 (MedchemExpress). After 24 h, Y27632 was withdrawn from the medium and cells were cultured for another 24 h. At day 0, cells were firstly induced by macrophage differentiation basal medium (SFD-M) which is RPMI 1640 medium supplemented with 2% B27 (Thermo Fisher Scientific), 1% L-GlutaMAX-I and 50 μg/ml ascorbic acid (Sigma Aldrich) and 10 ng/ml BMP4 (R&D Systems) for 24 h. Afterward, the medium was changed to SFD-M medium containing 10 ng/ml BMP4 and 2 μM GSK3 inhibitor CHIR99021 (Cayman Chemical) for another 48 h. At day 3, cells were replated onto Matrigel-coated dishes at a density of 4 × 10^4^ cells/ cm^2^ in SFD-M medium with 50 ng/ml VEGF (R&D Systems) and 10 ng ng/ml FGF2 (R&D Systems) for 48 h. At day 5, the medium was replaced with basal medium with 50 ng/ml VEGF, 10 ng ng/ml FGF2 and 10uM TGFβ inhibitor SB431542 (R&D Systems) for another 72 h. At day 8–10, floating cells were collected and medium was changed and supplemented with 50ng/ml M-CSF and 10ng/ml IL3 (R&D Systems) for another 3–5 days. From day 11–13 onward, the medium was changed to SFD-M medium with 50 ng/ml M-CSF for 3 days. All differentiation steps were cultured under normoxic conditions at 37 °C, 5% CO_2_. The protocol details are summarized in Figure S4A.

All embryonic stem cell studies were approved by the Institutional Review Board (IRB) at the University of Chicago, or by the Tri-Institutional ESCRO committee (Weill Cornell Medicine, Memorial Sloan Kettering Cancer Center, and Rockefeller University).

#### hPSC monocyte polarization

hPSC-derived CD14^+^ cells were plated on tissue culture plates at a density of 2×10^4^ cells/cm^2^ in SFD-M medium supplemented with 50 ng/mL M-CSF. After 2 days of culture, monocytes differentiated into M0 macrophages and polarized to M1 or M2 macrophages. For macrophages polarization, 100ng/mL LPS (Sigma-Aldrich) and 10ng/mL IFNγ (R&D Systems) were added for M1 induction, or 20 ng/m IL-4 (R&D Systems) was added for M2 induction in SFD-M medium supplemented with 50 ng/mL M-CSF, respectively. These cells were cultured for another three days before examination for expression of the M1 or M2 makers.

#### Giemsa Staining

Differentiating day 11–13 monocytes/macrophages were fixed on slides using Cytospin, followed by staining using Wright-Giemsa Stain (Sigma-Aldrich) according to the manufacturer’s instructions.

#### immunohistochemical staining

Histological study of lung tissues was performed on paraffin-embedded sections as previously described ^9^. For immunohistochemical staining, paraffin-embedded sections were deparaffinized and incubated with primary antibodies at 4°C overnight and secondary antibodies at room temperature for 1h. Primary antibodies and secondary antibodies are described in the supplementary Table. Nuclei were counterstained by Hoechst 33342 (Sigma). positive cells in lungs were randomly counted from different visions of slides by confocal microscopy. 12 views in each lung section were counted and averaged cell numbers per 0.04^2^ mm were used to define the distributions of positive cells in the lung tissues as described ^10^. Living cells in culture were directly fixed in 4% paraformaldehyde for 25 min, followed with 15 min permeabilization in 1 % triton X-100. For immunofluorescence, cells or tissue sections were immunostained with antibodies and counterstained with 4,6-diamidino-2-phenylindole (DAPI). Adjacent sections stained with H and E were used for comparison. The antibodies used for immunostaining or western blot experiments are listed in the key resource table.

#### Fluorescent activated cell sorting (FACS)

For FACS analysis, cells were resuspended in a FACS buffer (PBS with 0.1 % BSA and 2.5 mM EDTA). The cell suspension was then stained with PE-conjugated CD43 (Biolegend, clone MEM-59), APC-conjugated CD34 (BD, clone 581) to detect hematopoietic stem/progenitor cells (HSPC). PE-conjugated CD68 (Biolegend, clone Y1/82A), APC-conjugated CD11b (Biolegend, clone ICRF44), FITC-conjugated CD14 (Biolegend, clone HCD14) were used to detect monocyte/macrophages. Basically, cells were incubated with antibodies for 30 minutes at 4°C, followed with washed and suspended in 0.1 % BSA/PBS buffer. PE and APC filters were then used to detect cells double positive for CD43 and CD34 or CD68 and CD11b by signal intensity gating, FITC and APC were used to detect cells double positive for CD14 and CD11b. Negative controls stained with control IgG instead of primary antibodies were always performed with sample measurements. Flowcytometry machine of BD FACSAria II and software of Flowjo were mainly used to collect and analyze the flowcytometry data.

#### Cytokine analysis

Cytokines including hIL-1 β, IFN-α2, hIFN-γ, hTNF-α, hMCP-1, hIL-6, hIL-8, hIL-10, hIL-12p70, IL-17A, hIL-18, hIL-23, hIL-33 were detected according to the instruction of LEGENDplex™ kit (Biolegend, cat. no. 740808). In brief, 25ul supernatant was taken from the co-culture medium and mixed with 25 μl of premixed beads and 25 μl of detection antibodies. The mixtures were placed on a shaker at 400 r.p.m. for 2 h at RT. Then 25 μl of SA-PE was added to each tube and placed on a shaker at 500 r.p.m. for 30 min. The data were obtained by flow cytometry (FACSAria II, BD) and were analyzed with LEGENDplex v.8.0 (Biolegend).

#### SARS-CoV-2-Pseudo-Entry Viruses

Recombinant Indiana VSV (rVSV) expressing SARS-CoV-2 spikes was generated as previously described ^11–13^. HEK293T cells were grown to 80% confluency before transfection with pCMV3-SARS-CoV2-spike (kindly provided by Dr. Peihui Wang, Shandong University, China) using FuGENE 6 (Promega). Cells were cultured overnight at 37°C with 5% CO_2_. The next day, the media was removed and VSV-G pseudotyped ΔG-luciferase (G*ΔG-luciferase, Kerafast) was used to infect the cells in DMEM at an MOI of 3 for 1 hr before washing the cells with 1X DPBS three times. DMEM supplemented with 2% FBS and 100 I.U. /mL penicillin and 100 μg/mL streptomycin was added to the infected cells and they were cultured overnight as described above. The next day, the supernatant was harvested and clarified by centrifugation at 300xg for 10 min before aliquoting and storing at −80°C.

#### SARS-CoV-2 infections

SARS-CoV-2, isolate USA-WA1/2020 (NR-52281) was deposited by the Center for Disease Control and Prevention and obtained through BEI Resources, NIAID, NIH. SARS-CoV-2 was propagated in Vero E6 cells in DMEM supplemented with 2% FBS, 4.5 g/L D-glucose, 4 mM L-glutamine, 10 mM Non-Essential Amino Acids, 1 mM Sodium Pyruvate and 10 mM HEPES as described previously (insert citation for https://doi.org/10.1016/j.cell.2020.04.026).

hESC-derived lung and macrophage co-cultures in 96-well plates were infected with SARS-CoV-2 for 24 h at an MOI of 0.01 in the medium containing SFD:SFD-M=1:1. For immunofluorescence staining, cells were washed three times in PBS and fixed for 24 h in 5% formaldehyde for 24 h for immunofluorescent staining, prior to safe removal from the BSL-3 facility.

All work involving live SARS-CoV-2 was performed in the CDC/USDA-approved BSL-3 facility of the Global Health and Emerging Pathogens Institute at the Icahn School of Medicine at Mount Sinai in accordance with institutional biosafety requirements

#### SARS-CoV-2 entry virus infections.

To assay pseudo-typed virus infection, cells were seeded in 96 well plates. Pseudo-typed virus was added for MOI=0.01. At 2 hpi, the infection medium was replaced with fresh medium. At 24 hpi, cells were harvested for luciferase assay or immunohistochemistry analysis. For liver and lung organoids, organoids were seeded in 24-well plates, pseudo-typed virus was added for MOI=0.01 and centrifuged the plate at 1200g, 1 hour. At 24 hpi, organoids were fixed for immunohistochemistry or harvested for luciferase assay following the Luciferase Assay System protocol (E1501, Promega)

#### Single cell sequencing of hPSC-derived lung cells

Single-cell capture, reverse transcription, cell lysis, and library preparation was performed using the Single Cell 3’ version 3 kit and chip according to the manufacturer’s protocol (10x Genomics, USA). Single-cell suspensions were generated by dissociating the cultured RUES2 cells with 0.05% Trypsin/0.02% EDTA for 10–15 min, followed with passing through 40μM strainer. The single cell suspension was achieved through sorting the dissociated cells in flow cytometry singlets. Cell count was adjusted to 1000–2000 cells per ul to target an estimated capture of 8000 cells. Six input wells were used. Sequencing was performed on NovaSeq6000 with setting 28 for read 1 and 91 for read 2. The sequencing data were primarily analyzed by CellRanger pipeline v3.0.2 (10x Genomics, USA). In particular, raw fastq data were generated by CellRanger mkfastq; A custom reference genome was built by integrating the virus and luciferase sequences into the 10x pre-built human reference (GRCh38 v3.0.0) using CellRanger mkref. Alignment of the raw reads to the custom reference genome, removing duplicated transcripts using the unique molecular identifiers (UMIs) and assignment to single cells was performed using CellRanger count.

Briefly, we used cells Seurat 3.1.4 R package for data analysis and visualization ^1^. The Seurat object is required at least 200 and at most 6000 unique molecular identifiers (UMIs), genes detected (UMI count > 0) in less than two cells were removed. In addition, cells were excluded if more than 10% of sequences mapped to mitochondrial genes. In total, 5,080 cells from the sample passed these filters for quality.

Following the package suggestions, we used a linear model to mitigate the variation stemming from the number of detected unique molecules per cell. The differentially expressed genes were found by “vst” method and the top 3,000 differentially expressed genes were selected for PCA analysis. We used an elbow plot to determine the number of PCs. 20 PCs were used for each group of cells. Clustering resolution was set at 0.2. For co-culture analysis, Macrophages and lung cells were re-clustered and re-analyzed, respectively. Macrophages were integrated using the first 20 dimensions of PCs and clustering resolution was set at 0.1. UMAP plots, heatmaps, violin plots and dotplots were generated by the Seurat toolkit *FeaturePlot, DoHeatmap, VlnPlot* amd *DotPlot* functions, respectively. Cell types were determined using a combination of marker genes identified from the literature and the web-based tool Topp CellAtlas (https://toppgene.cchmc.org/)

### QUANTIFICATION AND STATSTICAL ANALYSIS

Sample sizes for all figures and tables were estimated based on our previous studies^4,5,14,15^. For each set of experiments, samples were prepared for all experimental arms at the same time. All statistical tests are 2-sided. No adjustments were made for multiple comparisons. The relevant investigators (FD, LG and LY) were blinded to experimental allocations among different experimental arms for all experiments. N=3 independent biological replicates were used for all experiments unless otherwise indicated. n.s. indicates a non-significant difference. For all parametric statistical analyses, data were determined to be normally distributed by the D’Agostino-Pearson test. For all parametric and non-parametric tests, variances were similar between groups being compared. For comparison between experimental and control groups at a specific time point or tissue site, 2-sided Student t-tests, one-way or two-way ANOVA test, chi-squared tests and two sided Kolmogorov-Smirnov test were used. All cells (RUES2, H1, HEK293T, THP-1, U937) were purchased from ATCC or WiCell in the past 2 years and were negative for mycoplasma. The hESC lines were regularly checked for chromosome abnormalities and maintained with normal chromosome numbers.

## Supplementary Material

Supplement

Supplement

Supplement

Supplement

Supplement

Supplement

Supplement

Supplement

Supplement

## Figures and Tables

**Table:** KEY RESOURCES TABLE

REAGENT or RESOURCE	SOURCE	IDENTIFIER
**Antibodies**		
Mouse monoclonal anti-CD68	eBioscience	#14-0688-82
Rabbit polyclonal anti-CD80	BOSTER	#A00196-1
CD80-PE, human	miltenyi Biotec	#130-117-683
CD206-PE, human	miltenyi Biotec	#130-095-220
Rabbit monoclonal anti-CD163	Abcam	#ab182422
Rabbit polyclonal anti-IL6	Affinity	#DF6087
Goat polyclonal anti-ACE2	R&D system	#AF933
PE-conjugated CD43	eBioscience	#eBio84-3C1
APC-conjugated CD34	BD Biosciences	clone 581
PE-conjugated CD68	Biolegend	clone Y1/82A
APC-conjugated CD11b	Biolegend	clone ICRF44
FITC-conjugated CD14	Biolegend	clone HCD14
Anti-NKX2.1 Antibody	Seven Hills Bioreagents	#WRAB-1231
Anti-FOXA2 Antibody	Santa Cruz	#sc-6554
Anti-SOX2 Antibody	Santa Cruz	#sc-17320
Anti-SP-B Antibody	Seven Hills Bioreagents	#WRAB-48604
Anti-Pro-SP- C Antibody	Seven Hills Bioreagents	#WRAB-9337
Anti-FOXJ1 Antibody	Sigma-Aldrich	#HPA005714-1
Firefly luciferase Monoclonal Antibody (CS 17)	Thermo Fisher Scientific	#35-6700
Recombinant Anti-Firefly Luciferase antibody	Abcam	#ab185924
Mouse Anti-SARS-CoV-Spike antibody	Provided by Dr. Tom Moran	2B3E5
Donkey anti-Mouse IgG (H+L) Highly Cross-Adsorbed Secondary Antibody, Alexa Fluor 488	Thermo Fisher Scientific	#A-21202
Alexa Fluor 488 AffiniPure Donkey Anti-Guinea Pig IgG (H+L)	Jackson Immunoresearch Labs	#706-545-148
Donkey anti-Mouse IgG (H+L) Highly Cross-Adsorbed Secondary Antibody, Alexa Fluor 594	Thermo Fisher Scientific	#A-21203
Donkey anti-Rabbit IgG (H+L) Secondary Antibody, Alexa Fluor 594 conjugate	Thermo Fisher Scientific	#A-21207
Donkey anti-Rabbit IgG (H+L) Secondary Antibody, Alexa Fluor 647 conjugate	Thermo Fisher Scientific	#A-31573
Donkey anti-Mouse IgG (H+L) Secondary Antibody, Alexa Fluor 647	Thermo Fisher Scientific	#A-31571
Donkey anti-Goat IgG (H+L) Cross-Adsorbed Secondary Antibody, Alexa Fluor 647	Thermo Fisher Scientific	#A-21447
Donkey antiChicken IgG (H+L) Cross-Adsorbed Secondary Antibody, Alexa Fluor 488	Jackson Immunoresearch Labs	#703-545-155
Donkey anti-Sheep IgG (H+L) Cross-Adsorbed Secondary Antibody, Alexa Fluor 647	Thermo Fisher Scientific	#A-21448
**Chemicals, Peptides, and Recombinant Proteins**		
Activin A	R&D Systems	#338-AC-500/CF
Y-27632	MedchemExpress	#HY-10583
Recombinant Human BMP-4 Protein	R & D Systems	#314-BP
Recombinant Human bFGF	R&D Systems	#233-FB-500
Dorsomorphin dihydrochloride	R&D Systems	#3093/50
SB431542	R&D Systems	#1614/50
IWP2	R&D Systems	#3533/50
CHIR99021	Cayman Chemical	#13122
Recombinant Human FGF-10 Protein	R&D Systems	#345-FG-250
Recombinant Human KGF/FGF-7 Protein	R&D Systems	#251-KG-01M
Retinoic acid	Sigma-Aldrich	#R2625
Dexamethasone	Sigma-Aldrich	#D4902
8-Bromo-cAMP	Sigma-Aldrich	#B5386
IBMX	Sigma-Aldrich	#15879
Recombinant Human VEGF Protein	R&D Systems	#293-VE-500/CF
Recombinant Human IL-3 Protein	R&D Systems	#203-IL-050/CF
Recombinant Human M-CSF Protein	R&D Systems	#216-MC-025
Recombinant Human IL4 Protein	R&D Systems	#204-IL-050
IFNγ	R&D Systems	#285-IF-100
LPS	Sigma-Aldrich	#L4391-1MG
DAPI	Santa Cruz	#sc-3598
Hoechst 33342	Sigma-Aldrich	# B2261-100mg
Wright-Giemsa Stain	Sigma-Aldrich	#WG16-500ML
**Culture Medium**		
F12	Gibco Thermo Fisher	#31765035
β-mercaptoethanol	Sigma Aldrich	#M3148
Penicillin- Streptomycin (5,000 U/mL)	Gibco Thermo Fisher	#15070063
MEM Non-Essential Amino Acids Solution (100X)	Gibco Thermo Fisher	#11140050
IMDM	Gibco Thermo Fisher	#21056023
GlutaMAX Supplement	Thermo Fisher Scientific	#35050079
Accutase	Stemcell Technologies	#07920
Matrigel	Corning	#354234
Fibronectin (FN)	Thermo Fisher Scientific	#356008
N2 supplement	Thermo Fisher Scientific	#17502-048
B27	Thermo Fisher Scientific	#12587-010
DMEM/F12	Thermo Fisher Scientific	#10565-018
Knockout serum replacement(KOSR)	Thermo Fisher Scientific	#10828-028
FBS	Gibco Thermo Fisher	#10099141C
Monothioglycerol	Sigma Aldrich	#M6145
Ascorbic acid	Sigma Aldrich	#A4403
Bovine serum albumin(BSA)	Sigma Aldrich	#A9418
**Experimental Models: Cell Lines**	Seven Hills Bioreagents	#WRAB-1231
hESC line H1	Harvard University	#0014
hESC line-RUES2	The Rockefeller University	#0013
HEK293T	ATCC	#CRL-11268
Vero E6	ATCC	#CRL-1586
THP-1	ATCC	#TIB-202
U937	ATCC	#CRL-1593.2
Mouse embryonic fibroblasts	Global Stem	#GSC-6001G
**Software and Algorithms**		
Cell Ranger	10X Genomics	https://support.10xgenomics.com/single-cell-gene-expression/software/overview/welcome
Scran	Lun ATL, McCarthy DJ, Marioni JC (2016). “A step-by-step workflow for low-level analysis of single-cell RNA- seq data with Bioconductor.” *F1000Res,* **5**, 2122. doi: 10.12688/f1000research.9501.2.	https://bioconductor.org/packages/release/bioc/html/scran.html
Rstudio	Rstudio	https://rstudio.com
Seurat R package v3.1.4	1	https://satijalab.org/seurat/
DAVID6.8	LHRI	https://david.ncifcrf.gov/home.jsp
Adobe illustrator CC2017	Adobe	https://www.adobe.com/product/photoshop.html
Graphpad Prism 8.0	Graphpad software	https://www.graphpad.com
LEGENDplex v8.0	Biolegend	https://www.biolegend.com/en-us/legendplex
FlowJo v x.0.7	BD Biosciences	https://www.flowjo.com/
ToppCell Atlas	Toppgene	https://toppgene.cchmc.org/
