# Supplementary material for: Modeling COVID-19 with Human Pluripotent Stem Cell-Derived Cells Reveals Synergistic Effects of Anti-inflammatory Macrophages with ACE2 Inhibition Against SARS-CoV-2
Source: Res Sq. 2020 Sep 15:rs.3.rs-62758. Originally published 2020 Aug 20. Preprint. [Version 2] doi: 10.21203/rs.3.rs-62758/v2 (PMC7444287; doi:10.21203/rs.3.rs-62758/v2)
Supplement: Supplement 1 [file NIHPPRS62758v2-supplement-1.pdf]

Supplementary Files

This is a list of supplementary files associated with this preprint. Click to download.

- FigureS1.jpg
- FigureS2.jpg
- FigureS3.jpg

- FigureS4.jpg
- FigureS5.jpg
- FigureS6.jpg
- FigureS7.jpg
- FigureS8.jpg
- FigureS9.jpg
